# Antibody response to pneumococcal and influenza vaccination in patients with rheumatoid arthritis receiving abatacept

**DOI:** 10.1186/s12891-016-1082-z

**Published:** 2016-05-26

**Authors:** Rieke Alten, Clifton O. Bingham, Stanley B. Cohen, Jeffrey R. Curtis, Sheila Kelly, Dennis Wong, Mark C. Genovese

**Affiliations:** Schlosspark-Klinik University Medicine, Berlin, Germany; Johns Hopkins University, Baltimore, MD USA; Metroplex Clinical Research Center, Dallas, TX USA; University of Alabama at Birmingham, Birmingham, AL USA; Bristol-Myers Squibb, Princeton, NJ USA; Stanford University, Palo Alto, CA USA; University Medicine Berlin, Berlin, 14059 Germany

**Keywords:** Abatacept, Influenza, Pneumococcal, Rheumatoid arthritis, Vaccination, Immunization

## Abstract

**Background:**

Patients with rheumatoid arthritis (RA), including those treated with biologics, are at increased risk of some vaccine-preventable infections. We evaluated the antibody response to standard 23-valent pneumococcal polysaccharide vaccine (PPSV23) and the 2011–2012 trivalent seasonal influenza vaccine in adults with RA receiving subcutaneous (SC) abatacept and background disease-modifying anti-rheumatic drugs (DMARDs).

**Methods:**

Two multicenter, open-label sub-studies enrolled patients from the ACQUIRE (pneumococcal and influenza) and ATTUNE (pneumococcal) studies at any point during their SC abatacept treatment cycle following completion of ≥3 months’ SC abatacept. All patients received fixed-dose abatacept 125 mg/week with background DMARDs. A pre-vaccination blood sample was taken, and after 28 ± 3 days a final post-vaccination sample was collected. The primary endpoint was the proportion of patients achieving an immunologic response to the vaccine at Day 28 among patients without a protective antibody level to the vaccine antigens at baseline (pneumococcal: defined as ≥2-fold increase in post-vaccination titers to ≥3 of 5 antigens and protective antibody level of ≥1.6 μg/mL to ≥3 of 5 antigens; influenza: defined as ≥4-fold increase in post-vaccination titers to ≥2 of 3 antigens and protective antibody level of ≥1:40 to ≥2 of 3 antigens). Safety and tolerability were evaluated throughout the sub-studies.

**Results:**

Pre- and post-vaccination titers were available for 113/125 and 186/191 enrolled patients receiving the PPSV23 and influenza vaccine, respectively. Among vaccinated patients, 47/113 pneumococcal and 121/186 influenza patients were without protective antibody levels at baseline. Among patients with available data, 73.9 % (34/46) and 61.3 % (73/119) met the primary endpoint and achieved an immunologic response to PPSV23 or influenza vaccine, respectively. In patients with pre- and post-vaccination data available, 83.9 % in the pneumococcal study demonstrated protective antibody levels with PPSV23 (titer ≥1.6 μg/mL to ≥3 of 5 antigens), and 81.2 % in the influenza study achieved protective antibody levels (titer ≥1:40 to ≥2 of 3 antigens) at Day 28 post-vaccination. Vaccines were well tolerated with SC abatacept with background DMARDs.

**Conclusions:**

In these sub-studies, patients with RA receiving SC abatacept and background DMARDs were able to mount an appropriate immune response to pneumococcal and influenza vaccines.

**Trial registration:**

NCT00559585 (registered 15 November 2007) and NCT00663702 (registered 18 April 2008).

**Electronic supplementary material:**

The online version of this article (doi:10.1186/s12891-016-1082-z) contains supplementary material, which is available to authorized users.

## Background

Patients with rheumatoid arthritis (RA), including those being treated with biologics, are at an increased risk of some vaccine-preventable infections [1–4]. Two of the most frequent infections that have resulted in increased hospitalizations and/or death among patients with RA are caused by *Streptococcus pneumoniae* and *Haemophilus influenzae*, for which vaccinations exist [1]. Thus, implementation of a vaccination strategy is needed for daily clinical practice [4].

Patients with RA may need to receive immunizations following initiation of biologic therapy if their immunization status is not up to date (e.g. pneumococcal vaccine, annual seasonal flu vaccine) [4–6]. Given concerns regarding infection in patients receiving biologic therapy, treatment guidelines recommend routine use of pneumococcal and influenza vaccines in immunocompromised patients [7]. However, despite recommendations, the use of vaccines (e.g. pneumococcal and non-live influenza vaccines) is low in patients with RA compared with the general population [8–10]. This is partly due to uncertainty regarding the safety and efficacy of vaccines in patients treated with immunomodulatory therapies [4, 11].

Abatacept is approved for the treatment of moderate-to-severe RA, as an intravenous (IV) weight-tiered dosing regimen, and as a subcutaneous (SC) fixed dose. Abatacept is a soluble fusion protein that selectively modulates the CD28:CD80/86 co-stimulatory signal required for full T-cell activation [12–15]. Long-term treatment with SC and IV abatacept is associated with low incidences of serious infections and is well tolerated [16, 17]. A previous study in healthy volunteers suggested that, although responses may be blunted, IV abatacept does not impair the ability to mount an appropriate immune response to the tetanus toxoid or 23-valent pneumococcal vaccines [18]. In a sub-study of the ARRIVE (Abatacept Researched in Rheumatoid arthritis patients with an Inadequate anti-TNF response to Validate Effectiveness) trial, 81 and 75 % of abatacept-treated patients with active RA responded to at least one pneumococcal or influenza strain, respectively, demonstrating that patients treated with abatacept are able to mount an immune response to pneumococcal or influenza vaccination [19, 20]. In addition, in patients with psoriasis treated with IV abatacept, responses to two T cell-dependent antigens to which the patients had not been previously exposed (neoantigens: PhiX174 and keyhole limpet hemocyanin) were reduced, but not completely blocked (Additional file 1: Table S1) [21]. In this report, we describe results from influenza and pneumococcal vaccination sub-studies that were performed in patients with RA receiving SC abatacept. These results confirm the IV formulation results in a larger subset of patients, and thereby inform vaccination and treatment decisions for patients with RA who are considering treatment with or currently receiving abatacept.

## Methods

### Study design

Two multicenter, open-label sub-studies of the Phase IIIb ACQUIRE (Abatacept Comparison of sub[QU]cutaneous versus intravenous in Inadequate Responders to methotrexatE) and ATTUNE (Abatacept in subjecTs who swiTch from intravenoUs to subcutaNeous thErapy) studies evaluated response to the 23-valent pneumococcal polysaccharide vaccine (PPSV23) and seasonal influenza vaccine for enrolled patients. ACQUIRE was a double-blind, double-dummy, 6-month study in 1457 patients with active RA and an inadequate response to methotrexate (MTX) [22]. ATTUNE was an open-label, single-arm, 12-month study in 123 patients with active RA that was previously refractory to either MTX or anti-tumor necrosis factor drugs and who switched to SC abatacept following ≥4 years of IV abatacept in the AIM (Abatacept in Inadequate responders to Methotrexate) or ATTAIN (Abatacept Trial in Treatment of Anti-TNF INadequate responders) clinical trials [23]. During ACQUIRE and ATTUNE, SC abatacept was given at a fixed dose of 125 mg/week, with background disease-modifying anti-rheumatic drugs (DMARDs). SC abatacept-treated patients from ACQUIRE or ATTUNE were enrolled in the vaccination sub-study at any point during their SC abatacept treatment cycle following completion of ≥3 months of SC abatacept. Exclusion criteria for both sub-studies included: patients with a history of known allergy or allergy to egg, chicken proteins, neomycin, formaldehyde, or octoxinol-9; and patients who had received a pneumococcal vaccination within 5 years or an influenza vaccination within 6 months for each sub-study, respectively, based on the rate of decline of response [24, 25]. The vaccination sub-studies consisted of an initial visit for pre-vaccination blood sample collection and vaccine administration, follow-up of 28 ± 3 days [26–28], and a final visit for post-vaccination blood sample collection. Antibody response to the pneumococcal vaccine was evaluated for patients in ACQUIRE and ATTUNE; response to the influenza vaccine was evaluated only in ACQUIRE.

### Assessments

For both the pneumococcal and influenza vaccination sub-studies, the primary endpoint was the proportion of patients without a protective antibody level to the vaccine antigens at baseline who achieved an immunologic response (defined below) to the vaccine at Day 28. Exploratory endpoints included immunologic responses in patients with protective vaccine antibodies at baseline, and the proportion of patients achieving post-vaccination protective antibody levels. Efficacy assessments were based on antibody response to the standard PPSV23 or trivalent seasonal influenza virus vaccine in adult patients who were on a stable dose of SC abatacept and background DMARD therapy. Antibody titers for the pneumococcal vaccine were evaluated using a standard enzyme-linked immunosorbent assay for the following five antigens: 9V, 14, 18C, 19F, and 23F (antigens previously investigated during pneumococcal vaccine studies [29–31]). For the influenza vaccine, antibody titers were evaluated for the following three antigens: H1N1 (A/California), A/H3N2 (A/Victoria), and B/Brisbane. The primary pre-specified efficacy endpoint for the pneumococcal sub-study was the proportion of patients achieving an immunologic response, defined as a ≥2-fold increase in post-vaccination titers to ≥3 of 5 antigens (9V, 14, 18C, 19F, and 23F) [18, 21, 23, 29, 30, 32] at Day 28, in patients without a protective antibody level to these antigens at baseline. A protective antibody response to the pneumococcal antigens was defined as a titer of ≥1.6 μg/mL to ≥3 to 5 antigens [19, 21, 23, 25, 30, 31]. A conservative immunologic response to the pneumococcal antigens (with respect to the standard definition used for the primary endpoint) was defined as a ≥4-fold increase in post-vaccination titers to ≥3 of 5 antigens (9V, 14, 18C, 19F, and 23F). The primary efficacy endpoint specified in the influenza sub-study was the proportion of patients achieving an immunologic response, defined as a ≥4-fold increase in post-vaccination titers to ≥2 of 3 evaluated 2011–2012 influenza antigens (A/H1N1, A/H3N2, and B/Brisbane) [20, 21, 23, 30, 33, 34] at Day 28, in patients without a protective antibody level to these antigens at baseline. A protective antibody response to the influenza antigens was defined as a titer of ≥1:40 to ≥2 of 3 influenza antigens [21, 23, 30, 31, 34]. A conservative immunologic response to influenza was not defined. Antibody titers for the influenza vaccine response were evaluated using a hemagglutination inhibition assay.

Safety and tolerability were assessed throughout the sub-studies. The following adverse event (AE) assessments were performed: all AEs including clinical and laboratory categorized by severity, serious AEs (SAEs), and AEs of special interest (i.e. infections, autoimmune disorders, injection reactions to the vaccine). Safety assessments also included discontinuations due to AEs, and AEs specific to the pneumococcal or influenza vaccine. AEs and SAEs were classified using the Medical Dictionary for Regulatory Activities.

### Statistical analyses

Patients with >42 days between the pre- and post-vaccination sample dates were excluded from all analyses. A logistic regression model (odds ratios [OR] and 95 % confidence intervals [CIs]) was performed to evaluate the relationship of baseline factors (i.e. protective antibody level, MTX dose, concomitant steroid use, and age) to the primary endpoint and the proportion of patients achieving protective antibody levels (concomitant steroid use only).

## Results

### Patient population

**Fig. 1:**
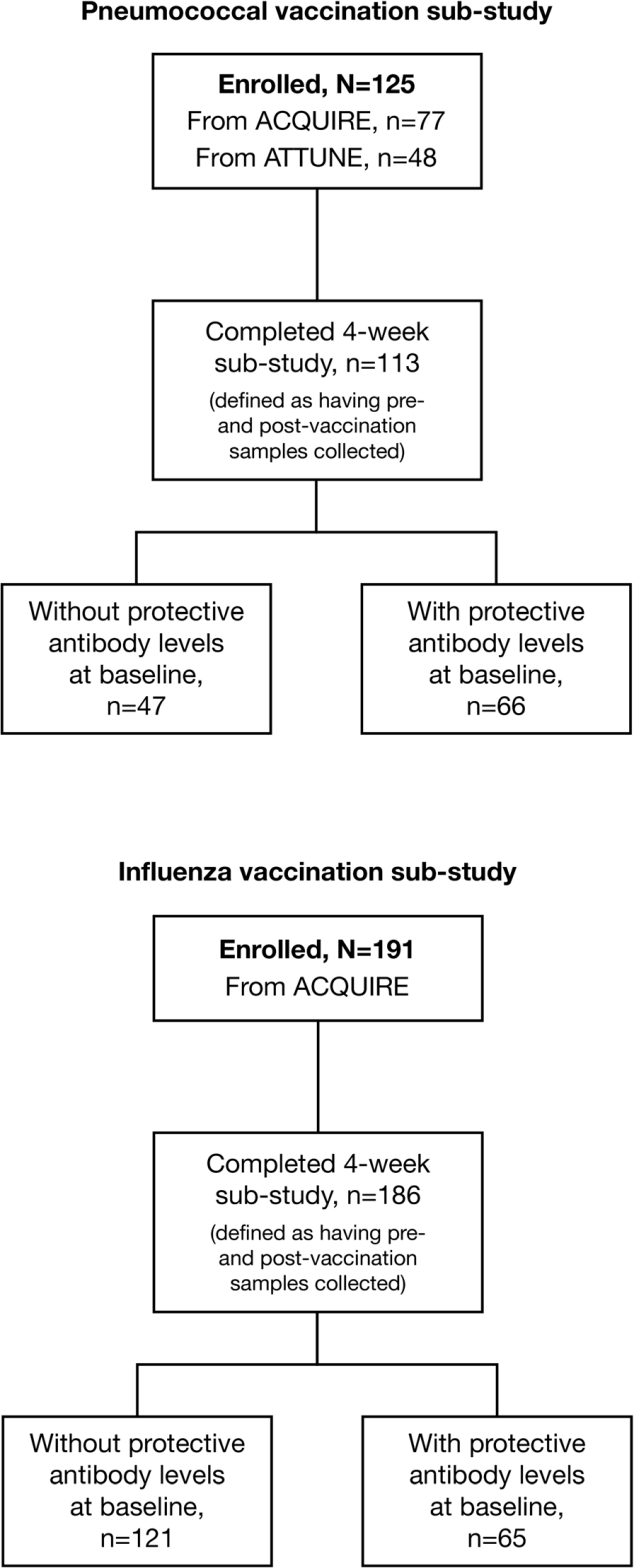
Disposition of patients in the pneumococcal and influenza vaccination sub-studies

A total of 125 patients (77 from ACQUIRE and 48 from ATTUNE) were enrolled in the pneumococcal vaccine sub-study and received the PPSV23 vaccination. Pre- and post-vaccination titers were available for 90.4 % of patients (113/125), and these patients represented those who completed the 4-week sub-study; 41.6 % of patients (47/113) were without protective antibody levels at baseline (Fig. 1). In the pneumococcal vaccine study, one patient had >42 days between pre- and post-vaccination samples (57 days) and was excluded from analyses.

A total of 191 patients from the ACQUIRE study were enrolled in the influenza vaccine sub-study and received the influenza vaccine. Of these patients, 97.4 % (186/191) had both pre- and post-vaccination titer samples collected and thus completed the 4-week sub-study; 65.1 % (121/186) were without protective antibody levels at baseline (Fig. 1). In the influenza vaccine sub-study, two patients had >42 days between pre- and post-vaccination samples (46 and 43 days) and were excluded from the analyses. Baseline demographics and clinical characteristics are shown in Tables 1 and 2.

**Table 1:** Baseline demographics and clinical characteristics for the pneumococcal vaccination population

Characteristic	Pneumococcal vaccine population (*N* = 125)
Age	
Mean (SD), years	45.7 (13.8)
Sex	
Women, *n* (%)	107 (85.6)
Men, *n* (%)	18 (14.4)
Race	
White, *n* (%)	124 (99.2)
Black/African American, *n* (%)	1 (0.8)
Region	
South America, *n* (%)	105 (84.0)
North America, *n* (%)	20 (16.0)
Weight	
Mean (SD), kg	68.9 (17.9)
Duration of abatacept exposure during main study	
Mean (SD), months	26.4 (2.5)
Tender joint count/28	
Mean (SD)	20.4 (16.7)
Swollen joint count/28	
Mean (SD)	13.9 (11.5)
Patient pain	
100-mm VAS, mean (SD)	51.0 (29.4)
HAQ-DI	
Mean (SD)	1.4 (0.8)
C-reactive protein	
Mean (SD), mg/dL	1.8 (2.5)
Patient global assessment	
100-mm VAS, mean (SD)	43.9 (31.9)
Physician global assessment	
100-mm VAS, mean (SD)	48.5 (26.3)
DAS28 (C-reactive protein)	
Mean (SD)	5.0 (1.9)
Concomitant methotrexate	
*n* (%)	115 (92.0)

*DAS28* Disease Activity Score 28, *HAQ-DI* Health Assessment Questionnaire-Disability Index, *SD* standard deviation, *VAS* visual analog scale

**Table 2:** Baseline demographics and clinical characteristics for the influenza vaccination population

Characteristic	Influenza vaccine population (*N* = 191)
Age	
Mean (SD), years	44.9 (12.6)
Sex	
Women, *n* (%)	172 (90.1)
Men, *n* (%)	19 (9.9)
Race	
White, *n* (%)	188 (98.4)
Black/African American, *n* (%)	2 (1.0)
Other, *n* (%)	1 (0.5)
Region	
South America, *n* (%)	156 (81.7)
North America, *n* (%)	35 (18.3)
Weight	
Mean (SD), kg	68.4 (17.3)
Duration of abatacept exposure during main study	
Mean (SD), months	37.6 (2.9)
Tender joint count/28	
Mean (SD)	30.7 (14.4)
Swollen joint count/28	
Mean (SD)	20.3 (8.0)
Patient pain	
100-mm VAS, mean (SD)	65.5 (22.2)
HAQ-DI	
Mean (SD)	1.7 (0.6)
C-reactive protein,	
Mean (SD), mg/dL	2.4 (2.7)
Patient global assessment	
100-mm VAS, mean (SD)	64.7 (21.5)
Physician global assessment, 100-mm VAS	
100-mm VAS, mean (SD)	60.3 (17.9)
DAS28 (C-reactive protein)	
Mean (SD)	6.3 (0.8)
Concomitant methotrexate	
*n* (%)	186 (97.4)

*DAS28* Disease Activity Score 28, *HAQ-DI* Health Assessment Questionnaire-Disability Index, *SD* standard deviation, *VAS* visual analog scale

### Efficacy

#### Immunologic responses to vaccination

In the pneumococcal vaccine population with non-protective antibody levels at baseline, 73.9 % of patients mounted a response to the pneumococcal vaccine by achieving a ≥2-fold increase in post-vaccination titers to ≥3 of 5 pneumococcal antigens assessed (Table 3). The proportion of patients who mounted a response to the influenza vaccine by achieving a ≥4-fold increase in post-vaccination titers to ≥2 of 3 influenza antigens was 61.3 % in patients without baseline protective antibody levels (Table 3). The proportion of patients with a response to vaccination was generally higher in patients without protective antibodies compared with patients with protective antibodies at baseline (Table 3). Overall, 55.4 % of patients in the pneumococcal sub-study demonstrated protective antibody levels (≥2-fold increase in post-vaccination titers to ≥3 of 5 antigens) at 28 days post-vaccination, and 49.5 % in the influenza sub-study demonstrated protective antibody levels (≥4-fold increase in post-vaccination titers to ≥2 of 3 antigens; Table 3).

**Table 3:** Immunologic responses to the pneumococcal and influenza vaccines at Day 28 post-vaccination

	Pneumococcal vaccine (*n* = 113)	Influenza vaccine (*n* = 186)
Primary definition		
Patients without protective antibody levels at baseline^a^		
n/N (%)	34/46 (73.9)	73/119 (61.3)
95 % CI	61.2, 86.6	52.6, 70.1
Patients with protective antibody levels at baseline		
n/N (%)	28/66 (42.4)	18/65 (27.7)
95 % CI	30.5, 54.3	16.8, 38.6
Total		
n/N (%)	62/112 (55.4)	91/184 (49.5)
95 % CI	46.2, 64.6	42.2, 56.7
Conservative definition		
Patients without protective antibody levels at baseline^a^		
n/N (%)	16/46 (34.8)	N/A
95 % CI	21.0, 48.5	
Patients with protective antibody levels at baseline		
n/N (%)	8/65 (12.3)	N/A
95 % CI	4.3, 20.3	
Total		
n/N (%)	24/111 (21.6)	N/A
95 % CI	14.0, 29.3	

*CI* confidence interval, *N/A* not applicable

^a^Primary endpoint. Patients with >42 days between the pre- and post-vaccination sample dates were excluded from the analysis. One patient from the pneumococcal study had >42 days between pre- and post-vaccination samples (57 days) and did not have protective antibody levels at baseline; the patient did not achieve an immunologic response post-vaccination. Two patients from the influenza study had >42 days between pre- and post-vaccination sample (46 days: patient did not have protective antibodies at baseline and did not achieve an immunologic response post-vaccination; 43 days: patient did not have protective antibodies at baseline and achieved immunologic response post-vaccination)

Analyses of the relationship of baseline factors to pneumococcal or influenza vaccine response showed that the presence of protective antibody titers at baseline was significantly associated with a lower likelihood of obtaining a response to vaccination (OR: 0.3 [95 % CI: 0.1, 0.6], *p* = 0.0013; OR: 0.2 [95 % CI: 0.1, 0.5], *p* < 0.0001, respectively; Table 4). Use of MTX, irrespective of dose, and concomitant corticosteroid use (mean [standard deviation] oral dose: pneumococcal, 6.8 [3.0] mg/day; influenza, 6.0 [2.6] mg/day) at sub-study baseline were not significant factors associated with response to the pneumococcal or influenza vaccine (Table 4). The results for these baseline factors (protective antibody titers, MTX dose, and steroids) in the total population were consistent, irrespective of age (Additional file 2: Table S2). However, baseline age on its own was significantly associated with vaccination response (Table 4). Younger age (<55 years) was associated with achieving a pneumococcal vaccine response, both in the subgroup of patients with protective antibody levels at baseline (OR: 2.7 [95 % CI 0.8, 8.6]) and in those without (OR: 3.3 [95 % CI: 0.8, 14.2]). Age <55 years was also associated with achieving an influenza vaccine response, both in the subgroup of patients with (OR: 2.4 [95 % CI: 0.5, 12.3] and without (OR: 3.0 [95 % CI: 1.3, 6.9]) protective antibody levels at baseline. Similar results were observed in the total population (OR: 2.8 [95 % CI: 1.2, 6.8], *p* = 0.0181; OR: 2.3 [95 % CI: 1.1, 4.7], *p* = 0.0211, respectively).

**Table 4:** Relationship of baseline factors with pneumococcal and influenza immunologic responses

Baseline characteristic		Pneumococcal vaccine		Influenza vaccine	
		n/N (%)	OR (95 % CI)	n/N (%)	OR (95 % CI)
Patients without protective antibody levels at baseline^a^					
MTX dose at baseline, mg/week	None	2/4 (50.0)	0.3 (0.0, 3.0)	1/3 (33.3)	0.4 (0.0, 4.5)
	>0–10	5/7 (71.4)	0.6 (0.1, 6.0)	17/25 (68.0)	1.6 (0.5, 4.7)
	>10–15	19/25 (76.0)	0.8 (0.1, 4.8)	35/36 (62.5)	1.3 (0.5, 3.0)
	>15	8/10 (80.0)		20/35 (57.1)	
Steroid at baseline	Yes	19/26 (73.1)	0.9 (0.2, 3.4)	47/74 (63.5)	1.3 (0.6, 2.7)
	No	15/20 (75.0)		26/45 (57.8)	
Age at main study baseline, years	<55	28/35 (80.0)	3.3 (0.8, 14.2)	60/88 (68.2)	3.0 (1.3, 6.9)
	≥55	6/11 (54.5)		13/31 (41.9)	
Patients with protective antibody levels at baseline^a^					
MTX dose at baseline, mg/week	None	2/6 (33.3)	0.6 (0.1, 4.1)	3/5 (60.0)	3.6 (0.5, 26.8)
	>0–10	8/15 (53.3)	1.4 (0.4, 5.4)	3/12 (25.0)	0.8 (0.2, 3.9)
	>10–15	9/25 (36.0)	0.7 (0.2, 2.3)	5/24 (20.8)	0.6 (0.2, 2.4)
	>15	9/20 (45.0)		7/24 (29.2)	
Steroid at baseline	Yes	16/37 (43.2)	1.1 (0.4, 2.9)	10/40 (25.0)	0.7 (0.2, 2.1)
	No	12/29 (41.4)		8/25 (32.0)	
Age at main study baseline, years	<55	23/47 (48.9)	2.7 (0.8, 8.6)	16/52 (30.8)	2.4 (0.5, 12.3)
	≥55	5/19 (26.3)		2/13 (15.4)	
Total population^b^					
Protective antibody level at baseline	Yes	28/66 (42.4) *	0.3 (0.1, 0.6)	18/65 (27.7) **	0.2 (0.1, 0.5)
	No	34/46 (73.9) *		73/119 (61.3) **	
MTX dose at baseline, mg/week	None	4/10 (40.0)	0.5 (0.1, 2.2)	4/8 (50.0)	1.2 (0.3, 5.2)
	>0–10	13/22 (59.1)	1.1 (0.4, 3.4)	20/37 (54.1)	1.4 (0.6, 3.2)
	>10–15	28/50 (56.0)	1.0 (0.4, 2.4)	40/80 (50.0)	1.2 (0.6, 2.3)
	>15	17/30 (56.7)		27/59 (45.8)	
Steroid at baseline	Yes	35/63 (55.6)	1.0 (0.5, 2.2)	57/114 (50.0)	1.1 (0.6, 1.9)
	No	27/49 (55.1)		34/70 (48.6)	
Age at main study baseline, years	<55	51/82 (62.2)	2.8 (1.2, 6.8) ***	76/140 (54.3)	2.3 (1.1, 4.7) ***
	≥55	11/30 (36.7)		15/44 (34.1)	

Based on the total population, regardless of protective antibody status at baseline. ORs and 95 % likelihood ratio CIs are based on a logistic regression model: vaccine response = baseline factor. ORs and CIs are presented for each category versus the reference category (baseline protective [no], MTX >15 mg/week, steroid [no], age ≥55 years). *P*-values are for confounding factors. Patients with >42 days between the pre- and post-vaccination sample dates were excluded from the analysis

*CI* confidence interval, *MTX* methotrexate, *OR* odds ratio

^a^Multivariate model; ^b^bivariate model; *p = 0.0013; **p < 0.0001; ***p < 0.05

#### Post-vaccination protective antibody levels

In patients without protective pneumococcal antibody levels at baseline, the geometric mean titers (GMTs) for pre-vaccination pneumococcal antibodies across the five individual antigens ranged from 0.42 to 1.29, and increased to 1.69–5.17 post-vaccination; 47.5–75.5 % of these patients achieved a ≥2-fold increase in antibody titer to each of the five antigens compared with baseline (Additional file 3: Table S3). In patients without protective influenza antibody levels at baseline, the pre-vaccination influenza GMT across the three individual antigens ranged from 15.8 to 41.8, and increased to 79.7–280.9 post-vaccination. The proportion of these patients who achieved a ≥4-fold increase in antibody titer to each of the three influenza antigens compared with baseline ranged from 42.0 to 68.3 % (Additional file 3: Table S3). In all patients, the pre-vaccination pneumococcal or influenza GMT across the individual antigens ranged from 1.1 to 3.7 and from 27.8 to 83.7, respectively, and increased to 2.9–9.7 or 99.0–296.1 post-vaccination (Additional file 4: Table S4). Immunologic responses for the total populations ranged from 43.8 to 57.1 % with the pneumococcal vaccination and from 35.3 to 54.9 % with the influenza vaccination (Additional file 4: Table S4).

A high proportion of patients without protective antibody levels at baseline achieved protective antibody levels to ≥3 of 5 pneumococcal vaccine antigens (65.2 %) or ≥2 of 3 influenza vaccine antigens (73.1 %). In the total pneumococcal vaccination population, 83.9 % of patients demonstrated protective levels (antibody titer ≥1.6 μg/mL) to ≥3 of 5 antigens assessed post-vaccination (Additional file 5: Table S5). In the total influenza vaccination population, 82.1 % of patients achieved protective levels (antibody titer ≥1:40) to ≥2 of 3 antigens post-vaccination (Additional file 5: Table S5).

The impact of concomitant MTX and steroid use on achieving post-vaccination protective antibody levels is shown in Additional file 6: Table S6. In both sub-studies, a high percentage of patients achieved protective antibody levels post-vaccination, regardless of baseline concomitant medication use.

### Safety

Overall, 15/125 (12.0 %) and 27/191 patients (14.1 %) reported AEs with the pneumococcal and influenza vaccine, respectively. One of the AEs reported in the pneumococcal vaccine study was a sore left arm (1/125 [0.8 %]) and was reported as mild in intensity; this was considered by the investigator to be unrelated to the pneumococcal vaccine. Overall, infections were reported in 7 patients (5.6 %): nasopharyngitis (*n* = 6; 4.8 %) and urinary tract infection (*n* = 1; 0.8 %). All infections were of mild intensity, with the exception of one event of moderate intensity (nasopharyngitis). In the influenza sub-study there was one SAE reported (1/191 [0.5 %]); a 71-year-old woman experienced severe chest pain that began after vaccination on Day 18 of the vaccine sub-study. The chest pain was treated and resolved within 3 days. There was no action taken with the study drug and, in the opinion of the investigator, the event was unlikely to be related to the study drug. Overall, nine patients (4.7 %) had an infection in the influenza sub-study. Infections reported for more than one patient included nasopharyngitis (*n* = 4; 2.1 %) and upper respiratory tract infection (*n* = 2; 1.0 %). There were no deaths or discontinuations owing to AEs in either sub-study.

## Discussion

The efficacy of the pneumococcal and influenza vaccines given during SC abatacept therapy was consistent with previous reports for IV abatacept; specifically, patients were still able to mount an immune response when on abatacept [18–20]. In the present two sub-studies of patients with RA receiving abatacept and background DMARDs, the majority of vaccinated patients without protective antibody levels at baseline achieved an immune response to the standard PPSV23 and influenza virus vaccines. Despite the absence of a control group of vaccinated patients who were not receiving abatacept, nor larger numbers of vaccinated patients receiving abatacept who did not also receive MTX, the level of response in these two vaccination sub-studies suggests that abatacept does not interfere significantly with T-cell presentation and sensitization of an antigen given as a vaccine. The proportion of patients with an overall response to vaccination was lower in patients who had protective antibody levels at baseline compared with patients who did not, indicating that the booster responses were of smaller magnitude than the primary vaccination responses in these sub-studies. Consistent with this observation, analyses of baseline factors with vaccine response showed protective antibody titers at baseline to be a significant factor impacting vaccine response.

SC abatacept demonstrated no new safety signals following vaccinations, and pneumococcal and influenza vaccinations during SC abatacept administration were well tolerated. The safety profile of SC abatacept following vaccinations was consistent with previous reports for IV abatacept [18, 20]. Additionally, vaccination with the PPSV23 and influenza vaccines was well tolerated by patients with RA treated with abatacept, and these results are consistent with other vaccination studies in patients receiving biologic therapies, specifically rituximab, adalimumab, and tocilizumab [1, 26, 27, 30, 35].

Although the US Food and Drug Administration provides guidelines for influenza vaccination studies [34] and van Assen et al. [6] have reported recommendations from the European League Against Rheumatism for vaccinations in adults with autoimmune inflammatory rheumatic diseases, there are currently no specific guidelines for vaccination studies in patients with RA treated with biologics [1]. Across various vaccination studies in patients with RA that were included in a systematic analysis, there was some variability in reporting immunologic responses, including measures of GMT, seroresponse (immunologic response), seroprotection (protective antibody levels), and seroconversion (Additional file 1: Table S1) [1]. Whereas definitions of an immunologic response and protective antibody levels for influenza were relatively consistent across these studies [1, 30, 31, 34], controversy remains regarding the definition of protective antibody levels for each pneumococcal serotype [23]. An immunologic response to the PPSV23 in patients with RA has been defined as a ≥2-fold increase in post-vaccination titers to ≥3 of 5 antigens (9V, 14, 18C, 19F, and 23F) [18, 29, 30, 32]. In two RA vaccination studies, the protective antibody level for pneumococcal serotypes has been reported as a titer of 1.6 μg/mL [25, 30, 31]. In several other studies, the consensus value has been reported as 1.3 μg/mL [25], but studies have used a value as low as 1.0 μg/mL [1, 25–27] or as high as 2.0 μg/mL [1, 25, 36–38]. The use of different vaccination response definitions across studies in patients with RA presents a challenge to clinicians who are making biologic treatment decisions.

In addition to the definition of response, the use of concomitant medications (MTX and/or prednisone) is another factor that might impact the results of immunologic response; in particular, MTX has been shown to affect pneumococcal vaccination response in a dose-dependent manner [39]. However, in the present pneumococcal and influenza vaccination sub-studies, there was no significant impact of concomitant use of MTX, irrespective of dose, or of concomitant corticosteroids on the proportion of patients achieving protective antibody levels in patients treated with abatacept.

Age can also be a factor that influences immunologic responses to vaccines [40–42]. Elderly individuals (>65 years) might have greater difficulty mounting a protective response to influenza and pneumococcal vaccines compared with younger adults [40–43]. In the present studies, higher proportions of patients aged <55 years achieved responses to pneumococcal or influenza vaccination.

As a result of the predefined exclusion criteria, which stated that patients with >42 days between the pre- and post-vaccination sample dates were excluded from all analyses, data from three patients were not analyzed: one patient from the pneumococcal vaccine sub-study and two from the influenza vaccine sub-study. The patient excluded from the pneumococcal vaccine study did not have protective antibody levels at baseline and did not achieve an immunologic response post-vaccination; it is unlikely that results from this patient would have greatly impacted the overall results. Of the two patients excluded from the influenza vaccine sub-study, one did not have protective antibodies at baseline and did not achieve an immunologic response post-vaccination, while the other did not have protective antibodies at baseline and achieved an immunologic response post-vaccination. As a consequence, the excluded results for influenza vaccination may have cancelled each other out and therefore would not have greatly affected the overall results.

There are several limitations to these sub-studies. The RA population in these sub-studies consisted of patients who were receiving abatacept; therefore, there were no patients with RA who were not receiving abatacept as a control group for comparison. ATTUNE comprised a single SC abatacept 125-mg/week treatment arm; ACQUIRE comprised an SC abatacept 125-mg/week treatment arm and an IV abatacept weight-tiered dose treatment arm (not analyzed here). In addition, almost all patients also received concomitant MTX (with the exception of 10 and 5 patients for the pneumococcal and influenza vaccine studies, respectively). Although there was no control group of placebo/MTX for direct comparison, overall immune response levels in this study were on a par with those from controlled studies that have used similar definitions for vaccine response [30, 31]. Also, due to the lack of a control group, we were unable to evaluate the effects of abatacept on the immunogenicity of PPSV23 by comparing patients with RA treated with abatacept versus placebo/MTX. Functional antibody activity as measured by opsonophagocytosis was also not investigated. With regard to the pneumococcal vaccine assessed, the analyses in this vaccination sub-study did not determine responses to the pneumococcal conjugate vaccine (13-valent pneumococcal conjugate vaccine [PCV13]) or to the sequence of pneumococcal vaccines (PPSV23 and PCV13 separated by time) recently recommended by the Advisory Committee on Immunization Practices/Centers for Disease Control and Prevention [44]. Additionally, for the pneumococcal vaccination analysis, response to the five antigens utilized in this and other vaccination studies does not guarantee response to the remaining antigens in the PPSV23 [29]. Given the different patient populations included, caution should be used when drawing comparisons from the ACQUIRE (pneumococcal and influenza vaccine) and ATTUNE (pneumococcal vaccine) sub-studies.

## Conclusions

These data suggest that the majority of patients with RA receiving SC abatacept treatment are able to mount an appropriate primary or booster immune response to either the pneumococcal or the influenza vaccine. Furthermore, immunizations with pneumococcal polysaccharide and inactivated influenza vaccines have a good safety profile in abatacept-treated patients. Achieving optimal responses may require vaccination before initiating abatacept, if feasible; however, patients should continue to receive primary, booster, or annual vaccines.

## Additional files

Additional file 1: Table S1. Glossary. Description of data: Glossary of vaccination terminology. (DOCX 28 kb) Additional file 2: Table S2. Relationship of baseline factors with pneumococcal and influenza immunologic responses stratified by age (<55 or ≥55 years). Description of data: Pneumococcal and influenza vaccine responses are shown for patients <55 and ≥55 years according to baseline factors. (DOCX 29 kb) Additional file 3: Table S3. Geometric mean titers and immunologic response to individual antigens 28 dayspost-vaccination. Description of data: Pre-and post-vaccination geometric mean titers and immunologic responses are shown for individual pneumococcal and influenza vaccine antigens in patients without protective antibody levels at baseline. (DOCX 29 kb) Additional file 4: Table S4. Geometric mean titers and immunologic response to individual antigens 28 days post-vaccination. Description of data: Pre-and post-vaccination geometric mean titers and immunologic responses are shown for individual pneumococcal and influenza vaccine antigens in the total patient population. (DOCX 29 kb) Additional file 5: Table S5. Patients achieving protective antibody levels^a^ 28 days post-vaccination. Description of data: Percentages of patients achieving protective antibody levels to individual pneumococcal and influenza antigens 28 days post-vaccination are shown. (DOCX 29 kb) Additional file 6: Table S6. Patients achieving post-vaccination protective antibody levels^a^ by concomitant medication use. Description of data: Percentages of patients achieving protective antibody levels to pneumococcal and influenza antigens 28 days post-vaccination, according to baseline concomitant medication use, are shown. (DOCX 28 kb)

## Abbreviations

ACQUIRE: Abatacept Comparison of sub[QU]cutaneous versus intravenous in Inadequate Responders to methotrexatE
AE: Adverse event
AIM: Abatacept in Inadequate responders to Methotrexate
ARRIVE: Abatacept Researched in Rheumatoid arthritis patients with an Inadequate anti-TNF response to Validate Effectiveness
ATTAIN: Abatacept Trial in Treatment of Anti-TNF INadequate responders
ATTUNE: Abatacept in subjecTs who swiTch from intravenoUs to subcutaNeous thErapy
CI: Confidence interval
CRP: C-reactive protein
DAS: Disease Activity Score
DMARD: Disease-modifying anti-rheumatic drug
GMT: Geometric mean titer
HAQ-DI: Health Assessment Questionnaire-Disability Index
IV: Intravenous
MTX: Methotrexate
OR: Odds ratio
PCV13: 13-valent pneumococcal conjugate vaccine
PPSV23: 23-valent pneumococcal polysaccharide vaccine
RA: Rheumatoid arthritis
SAE: Serious adverse event
SC: Subcutaneous
SD: Standard deviation
VAS: Visual analog scale
