# Supplementary material for: Antibody response to pneumococcal and influenza vaccination in patients with rheumatoid arthritis receiving abatacept
Source: BMC Musculoskelet Disord. 2016 May 26;17:231. doi: 10.1186/s12891-016-1082-z (PMC4880815; doi:10.1186/s12891-016-1082-z)
Supplement: Additional file 1: Table S1. — Glossary. Description of data: Glossary of vaccination terminology. (DOCX 28 kb) [file 12891_2016_1082_MOESM1_ESM.docx]

**Additional file 1**

**Table S1** Glossary

| **Term** | **Definition** |
| --- | --- |
| Seroconversion | Development of antibodies in blood serum as a result of infection or immunization [1] |
| Geometric mean titer | Parameter used to assess the response to a vaccination [2, 3] |
| Neoantigen | An immunogen to which the host has not been previously exposed [2] |
| Booster | Adjuvants and repeated exposure to a vaccination to induce an adequate protective immune response [1] |
| Primary immune response | Immune response that follows the first exposure to an antigen |

^1^Rahier JF, Moutschen M, Van Gompel A, Van Ranst M, Louis E, Segaert S, et al. Vaccinations in patients with immune-mediated inflammatory diseases. Rheumatology (Oxford). 2010;49:1815–27

^2^Orange JS, Ballow M, Stiehm ER, Ballas ZK, Chinen J, De La Morena M, et al. Use and interpretation of diagnostic vaccination in primary immunodeficiency: a working group report of the Basic and Clinical Immunology Interest Section of the American Academy of Allergy, Asthma & Immunology. J Allergy Clin Immunol. 2012;130:S1–24

^3^Food and Drug Administration Center for Biologics Evaluation and Research (FDA CBER). Guidance for industry: clinical data needed to support the licensure of seasonal inactivated influenza vaccines*.* 2007. [http://www.fda.gov/BiologicsBloodVaccines/GuidanceComplianceRegulatoryInformation/Guidances/Vaccines/ucm074794.htm] Accessed 08 Oct 2015.
