# Supplementary material for: Antibody response to pneumococcal and influenza vaccination in patients with rheumatoid arthritis receiving abatacept
Source: BMC Musculoskelet Disord. 2016 May 26;17:231. doi: 10.1186/s12891-016-1082-z (PMC4880815; doi:10.1186/s12891-016-1082-z)
Supplement: Additional file 2: Table S2. — Relationship of baseline factors with pneumococcal and influenza immunologic responses stratified by age (<55 or ≥55 years). Description of data: Pneumococcal and influenza vaccine responses are shown for patients <55 and ≥55 years according to baseline factors. (DOCX 29 kb) [file 12891_2016_1082_MOESM2_ESM.docx]

**Additional file 2**

**Table S2** Relationship of baseline factors with pneumococcal and influenza immunologic responses stratified by age (<55 or ≥55 years)

|  | | Pneumococcal vaccine | | | | Influenza vaccine | | | |
| --- | --- | --- | --- | --- | --- | --- | --- | --- | --- |
|  |  | n/N (%) | | OR (95% CI) | | n/N (%) | | OR (95% CI) | |
| Stratified by age, years | | <55 | ≥55 | <55 | ≥55 | <55 | ≥55 | <55 | ≥55 |
| Protective antibody level at baseline | Yes No | 23/47 (48.9) 28/35 (80.0) | 5/19 (26.3) 6/11 (54.5) | 0.2 (0.1, 0.7) | 0.3 (0.1, 1.4) | 16/52 (30.8) 60/88 (68.2) | 2/13 (15.4) 13/31 (41.9) | 0.21 (0.1, 0.4) | 0.25 (0.1, 1.3) |
| MTX dose at baseline, mg/week | None >0–10 >10–15 >15 | 2/4 (50.0) 10/17 (58.8) 24/26 (66.7) 15/25 (60.0) | 2/6 (33.3) 3/5 (60.0) 4/14 (28.6) 2/5 (40.0) | 0.7 (0.1, 5.5) 1.0 (0.3, 3.3) 1.3 (0.5, 3.8) | 0.8 (0.1, 8.8) 2.3 (0.2, 28.2) 0.6 (0.1, 5.1) | 4/7 (57.1) 17/30 (56.7) 35/63 (55.6) 20/40 (50.0) | 0/1 (0.0) 3/7 (42.9) 5/17 (29.4) 7/19 (36.8) | 1.3 (0.3, 6.7) 1.3 (0.5, 3.4) 1.3 (0.6, 2.8) | 0.0 (0.0, 0.0) 1.3 (0.2, 7.5) 0.7 (0.2, 2.9) |
| Steroid at baseline | Yes No | 27/47 (57.4) 24/35 (68.6) | 8/16 (50.0) 3/14 (21.4) | 0.6 (0.3, 1.6) | 3.7 (0.7, 18.3) | 48/90 (53.3) 28/50 (56.0) | 9/24 (37.5) 6/20 (30.0) | 0.9 (0.5, 1.8) | 1.4 (0.4, 5.0) |

*CI* confidence interval, *MTX* methotrexate, *OR* odds ratio
