# Supplementary material for: Antibody response to pneumococcal and influenza vaccination in patients with rheumatoid arthritis receiving abatacept
Source: BMC Musculoskelet Disord. 2016 May 26;17:231. doi: 10.1186/s12891-016-1082-z (PMC4880815; doi:10.1186/s12891-016-1082-z)
Supplement: Additional file 3: Table S3. — Geometric mean titers and immunologic response to individual antigens 28 dayspost-vaccination. Description of data: Pre-and post-vaccination geometric mean titers and immunologic responses are shown for individual pneumococcal and influenza vaccine antigens in patients without protective antibody levels at baseline. (DOCX 29 kb) [file 12891_2016_1082_MOESM3_ESM.docx]

**Additional file 3**

**Table S3** Geometric mean titers and immunologic responses to individual antigens 28 days post-vaccination in patients without protective antibody levels at baseline

| Vaccine | Antigen | Pre-vaccination GMT  (95% CI) | Post-vaccination GMT  (95% CI) | Patients mounting an immunologic response^a^,  n/N (%) (95% CI) |
| --- | --- | --- | --- | --- |
| Pneumococcal^b^ | 9V | 0.71 (0.49, 1.03) | 2.37 (1.59, 3.54) | 40/53 (75.5) (63.9, 87.1) |
|  | 14 | 0.73 (0.46, 1.15) | 3.53 (1.98, 6.28) | 30/45 (66.7) (52.9, 80.4) |
|  | 18C | 1.29 (0.93, 1.78) | 5.17 (3.46, 7.70) | 23/34 (67.6) (51.9, 83.4) |
|  | 19F | 0.93 (0.66, 1.30) | 2.52 (1.58, 4.01) | 19/40 (47.5) (32.0, 63.0) |
|  | 23F | 0.42 (0.33, 0.55) | 1.69 (1.14, 2.49) | 43/66 (65.2) (53.7, 76.6) |
| Influenza^c^ | A/H1N1 | 19.3 (13.3, 25.4) | 280.9 (191.4, 370.4) | 84/123 (68.3) (60.1, 76.5) |
|  | A/H3N2 | 41.8 (26.7, 56.9) | 272.6 (169.2, 375.9) | 64/95 (67.4) (57.9, 76.8) |
|  | B/Brisbane | 15.8 (13.1, 18.6) | 79.7 (60.9, 98.5) | 55/131 (42.0) (33.5, 50.4) |

*CI* confidence interval, *GMT* geometric mean titer

^a^Defined as ≥2-fold increase in pneumococcal antibody titer versus baseline, or ≥4-fold increase in antigen antibody titer versus baseline. Patients with >42 days between the pre- and post-vaccination sample dates were excluded from the analysis; ^b^n = 47 for GMT analysis; ^c^n = 119 for GMT analysis
