# Supplementary material for: Antibody response to pneumococcal and influenza vaccination in patients with rheumatoid arthritis receiving abatacept
Source: BMC Musculoskelet Disord. 2016 May 26;17:231. doi: 10.1186/s12891-016-1082-z (PMC4880815; doi:10.1186/s12891-016-1082-z)
Supplement: Additional file 4: Table S4. — Geometric mean titers and immunologic response to individual antigens 28 days post-vaccination. Description of data: Pre-and post-vaccination geometric mean titers and immunologic responses are shown for individual pneumococcal and influenza vaccine antigens in the total patient population. (DOCX 29 kb) [file 12891_2016_1082_MOESM4_ESM.docx]

**Additional file 4**

**Table S4** Geometric mean titers and immunologic response to individual antigens 28 days post-vaccination in the total population

| Vaccine | Antigen | Pre-vaccination GMT Mean (95% CI) | Post-vaccination GMT Mean (95% CI) | Patients mounting immunologic response^a^ n/N (%)  (95% CI) |
| --- | --- | --- | --- | --- |
| Pneumococcal | 9V | 1.7 (1.3, 2.3) | 4.2 (3.2, 5.4) | 58/112 (51.8) (42.5, 61.0) |
|  | 14 | 2.2 (1.6, 3.1) | 7.0  (5.2, 9.5) | 56/111 (50.5) (41.1, 59.8) |
|  | 18C | 3.7  (2.9, 4.9) | 9.7 (7.7, 12.2) | 64/112 (57.1) (48.0, 66.3) |
|  | 19F | 2.2 (1.7, 2.9) | 5.0 (3.8, 6.6) | 49/112 (43.8) (34.6, 52.9) |
|  | 23F | 1.1 (0.9, 1.5) | 2.9 (2.3, 3.7) | 58/112 (51.8) (42.5, 61.0) |
| Influenza | A/H1N1 | 40.6 (31.2, 50.0) | 295.9  (221.7, 370.0) | 101/184 (54.9) (47.7, 62.1) |
|  | A/H3N2 | 83.7  (52.6, 114.8) | 296.1 (211.6, 380.6) | 96/184 (52.2) (45.0, 59.4) |
|  | B/Brisbane | 27.8 (23.3, 32.3) | 99.0 (78.5, 119.6) | 65/184 (35.3) (28.4, 42.2) |

*CI* confidence interval, *GMT* geometric mean titer

^a^Defined as ≥2-fold increase in pneumococcal antigen antibody titer versus baseline, or ≥4-fold increase in influenza antigen antibody titer versus baseline. Patients with >42 days between the pre- and post-vaccination sample dates were excluded from the analysis
