# Supplementary material for: Antibody response to pneumococcal and influenza vaccination in patients with rheumatoid arthritis receiving abatacept
Source: BMC Musculoskelet Disord. 2016 May 26;17:231. doi: 10.1186/s12891-016-1082-z (PMC4880815; doi:10.1186/s12891-016-1082-z)
Supplement: Additional file 5: Table S5. — Patients achieving protective antibody levelsa 28 days post-vaccination. Description of data: Percentages of patients achieving protective antibody levels to individual pneumococcal and influenza antigens 28 days post-vaccination are shown. (DOCX 29 kb) [file 12891_2016_1082_MOESM5_ESM.docx]

**Additional file 5**

**Table S5** Patients achieving protective antibody levels^a^ 28 days post-vaccination

| Vaccine | Antigen | Patients without protective antibody levels at baseline N = 47 n/N (%)  (95% CI) | Total population N = 113 n/N (%)  (95% CI) |
| --- | --- | --- | --- |
| Pneumococcal | ≥3 of 5 antigens | 30/46 (65.2) (51.5, 79.0) | 94/112 (83.9) (77.1, 90.7) |
|  | 9V | 29/46 (63.0) (49.1, 77.0) | 85/112 (75.9) (68.0, 83.8) |
|  | 14 | 28/46 (60.9) (46.8, 75.0) | 88/112 (78.6) (71.0, 86.2) |
|  | 18C | 36/46 (78.3) (66.3, 90.2) | 100/112 (89.3) (83.6, 95.0) |
|  | 19F | 28/46 (60.9) (46.8, 75.0) | 86/112 (76.8) (69.0, 84.6) |
|  | 23F | 28/46 (60.9) (46.8, 75.0) | 84/112 (75.0) (67.0, 83.0) |
| Influenza | ≥2 of 3 antigens | 87/119 (73.1) (65.1, 81.1) | 151/184 (82.1) (76.5, 87.6) |
|  | A/H1N1 | 85/119 (71.4) (63.3, 79.5) | 148/184 (80.4) (74.7, 86.2) |
|  | A/H3N2 | 96/119 (80.7) (73.6, 87.8) | 157/184 (85.3) (80.2, 90.4) |
|  | B/Brisbane | 69/119 (58.0) (49.1, 66.9) | 126/184 (68.5) (61.8, 75.2) |

*CI* confidence interval

^a^Defined as an antibody titer ≥1.6 µg/mL for pneumococcal antigens and ≥1:40 for influenza antigens. Patients with >42 days between the pre- and post-vaccination sample dates were excluded from the analysis. One patient from the pneumococcal study had >42 days between pre- and post-vaccination samples (57 days) and did not have protective antibody levels at baseline; the patient achieved an immunologic response post-vaccination. Two patients from the influenza study had >42 days between pre- and post-vaccination sample (46 days: patient did not have protective antibodies at baseline and did not achieve an immunologic response post-vaccination; 43 days: patient did not have protective antibodies at baseline and achieved an immunologic response post-vaccination)
