# Supplementary material for: Antibody response to pneumococcal and influenza vaccination in patients with rheumatoid arthritis receiving abatacept
Source: BMC Musculoskelet Disord. 2016 May 26;17:231. doi: 10.1186/s12891-016-1082-z (PMC4880815; doi:10.1186/s12891-016-1082-z)
Supplement: Additional file 6: Table S6. — Patients achieving post-vaccination protective antibody levelsa by concomitant medication use. Description of data: Percentages of patients achieving protective antibody levels to pneumococcal and influenza antigens 28 days post-vaccination, according to baseline concomitant medication use, are shown. (DOCX 28 kb) [file 12891_2016_1082_MOESM6_ESM.docx]

**Additional file 6**

**Table S6** Patients achieving post-vaccination protective antibody levels^a^ by concomitant medication use

| Concomitant medication use at baseline | | Pneumococcal vaccine n = 113 | | Influenza vaccine n = 186 | |
| --- | --- | --- | --- | --- | --- |
|  |  | n/N (%) | OR (95% CI) | n/N (%) | OR (95% CI) |
| MTX | None | 9/10 (90.0) | 1.4 (0.1, 14.1) | 6/8 (75.0) | 0.6 (0.1, 3.5) |
|  | >0–10 mg/week | 18/22 (81.8) | 0.7 (0.2, 3.1) | 31/37 (83.8) | 1.1 (0.3, 3.2) |
|  | >10–15 mg/week | 41/50 (82.0) | 0.7 (0.2, 2.5) | 65/80 (81.3) | 0.9 (0.4, 2.1) |
|  | >15 mg/week | 26/30 (86.7) |  | 49/59 (83.1) |  |
| Steroids | Yes | 50/63 (79.4) | 0.4 (0.1, 1.3) | 95/114 (83.3) | 1.3 (0.6, 2.7) |
|  | No | 44/49 (89.8) |  | 56/70 (80.0) |  |

Based on the total population, regardless of protective antibody status at baseline

*CI* confidence interval, *MTX* methotrexate, *OR* odds ratio

^a^Defined as an antibody titer ≥1.6 µg/mL for pneumococcal antigens and ≥1:40 for influenza antigens. OR and the 95% likelihood ratio CI are based on a logistic regression model: protective antibody levels at Day 28 = baseline factor. ORs and CIs are presented for each category versus the reference category (steroid [no], MTX >15 mg/week). Patients with >42 days between the pre- and post-vaccination sample dates were excluded from the analysis
